# Renal vascular patterns assessment before robot-assisted partial nephrectomy: a video-based correlation with hilum dissection

**DOI:** 10.1007/s00345-025-05701-6

**Published:** 2025-05-31

**Authors:** O. S. Barnoiu, H. R. Yazdani, A. V. Andersen

**Affiliations:** 1 Department of Urology, Sorlandet Hospital, Kristiansand, Norway; 2 Department of Urology, Sorlandet Hospital, Arendal, Norway

**Keywords:** Partial nephrectomy, Vascular pattern, Renal hilum dissection, Surgical video

## Abstract

**Purpose:**

Surgical video review (SVR) is an emerging tool for assessing patient outcomes, especially in complex surgeries such as robot-assisted partial nephrectomy (RAPN). Adhesive probability and morphological scores are used to evaluate fat management and warm ischemia, respectively; however, the factors influencing renal hilum control (RHC) during RAPN have not yet been assessed. The aim of this study is to use SVR to identify the renal vascular patterns and factors that influence RHC.

**Methods:**

We evaluated 60 surgical video recordings of patients undergoing RAPN in 2023–2024, and measured the time to hilum control (THC) and total operation time (TOT) using a stopwatch. Patient and surgical factors were recorded and SPSS software was used to identify the correlation of these factors and vascular patterns with THC.

**Results:**

We observed a median THC of 22.7 min representing 15.1% of TOT. A significant correlation was found between previous renal surgery (p = 0.033), complex vascular anatomy on the right side (artery bifurcation behind IVC) or left side (more than one artery) (p = 0.02) and a longer THC. No significant difference was found between surgeons (p = 0.753) or surgical approach (transperitoneal vs. retroperitoneal, p = 0.87).

**Conclusion:**

THC represents a relatively short part of the total RAPN time. Previous renal surgery and a complex vascular pattern with artery bifurcation behind IVC on the right side and more than one main renal artery on the left side, can lead to longer THC. A detailed understanding of renal vascular patterns can provide a patient-specific surgical planning and optimise strategies for RAPN.

## Introduction

Minimally invasive partial nephrectomy (PN), whenever feasible, is the standard of care for patients diagnosed with T1a and T1b kidney cancer [1]. However, this approach is technically challenging and requires a detailed understanding of renal and tumour surgical anatomy to optimise oncological, functional and perioperative outcomes [2].

Contrast-enhanced computer tomography (CT) of the abdomen is the reference standard for primary imaging of renal tumours and the vascular system. Study the renal arterial anatomy via CT scan has been previously used for arterial tree representation [3]. In approximately 75% of cases, a single renal artery and vein are present, but a high percentage (15–20%) of patients also show anatomic variations such as duplication or early branching [2]. Disposition of the renal hilar structures and their numbers are significantly more variable than the classical pattern given in standard textbooks of anatomy, and a precise knowledge of both normal and variant anatomies of renal hilum is essential in the era of robotic surgery [4].

Robotic surgery platforms enable high-quality video recordings, providing greater magnification and closer views of anatomical details. Surgical video review (SVR) is an emerging tool for assessing patient outcomes [5], especially in complex surgeries such as robot-assisted PN (RAPN). Three key surgical phases are critical during RAPN: renal hilum control (RHC), fat management and warm ischemia time (WIT). Adhesive probability and morphological scores are respectively used to evaluate fat management [6] and warm ischemia [7], along with SVR; however, the factors influencing RHC during RAPN have not yet been assessed.

The aim of this study is to use SVR to identify the factors that influence RHC according to the renal vascular patterns identified via renal CT scan.

## Material and methods

The storage of recorded surgical videos for a period of time is mandatory in Norway to ensure quality of healthcare services and provide medicolegal evidence. This requirement allowed us to retrospectively review surgical video recordings of 60 consecutive patients undergoing RAPN at our institution between January 2023 and June 2024. All procedures were performed on T1a tumours using Intuitive Xi robotic systems (Intuitive™, Sunnyvale, CA, USA). As part of the kidney cancer surgical team, one surgeon (O.B.) reviewed the video recordings and measured time to hilum control (THC) and total operation time (TOT) using a digital stopwatch and exact visual cues for starting and ending points. For THC, we defined the starting point as when the monopolar-curved scissors’ cut function is first used to dissect the renal hilum (gonadal vein on the left side, inferior vena cava (IVC) on the right side), right after colon decollation for the transperitoneal approach or the posterior opening of Gerota’s fascia, for the retroperitoneal approach. The ending point for THC was defined as when the Hem-o-lock clip is applied to the vessel loop surrounding the renal artery. In cases of more than one artery or the need to clamp the renal vein, the ending point was defined as when the Hem-o-lock clip is applied to the vessel loop surrounding the last vascular structure of the hilum. The TOT compromised the time from the first to the last use of the robotic instruments.

**Fig. 1:**
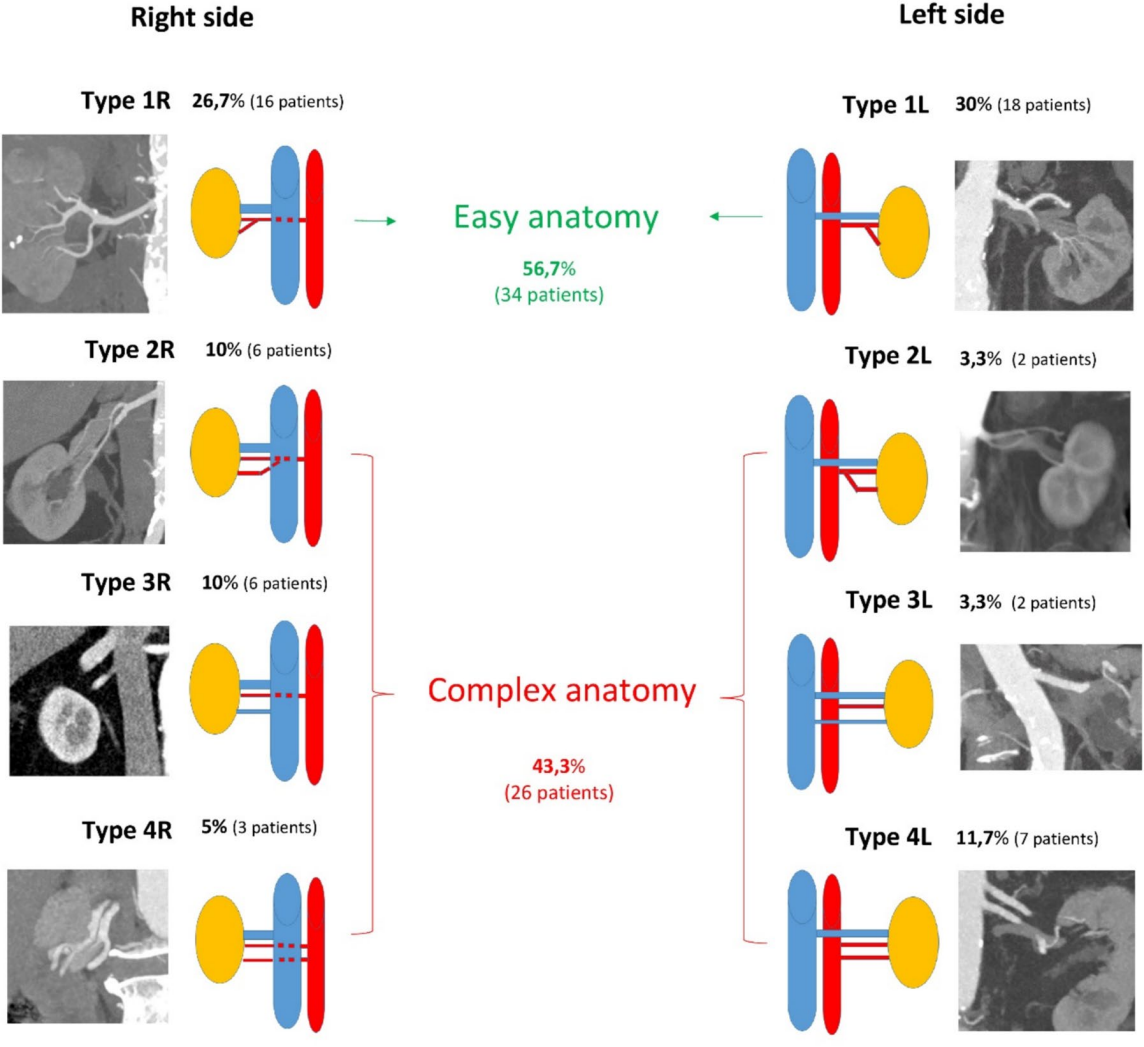
Vascular patterns identified on preoperative CT scan

We assessed the renal vascular patterns using evaluation of the arterial and venous phase of the CT scans, performed preoperatively. Eight different vascular patterns (Fig. 1) were identified according to side, four on the right (Type 1R, 2R, 3R and 4R) and four on the left (Type 1L, 2L, 3L and 4L). Also the number of arteries or veins and proximity of the artery bifurcation to aorta were taken into consideration. We defined the anatomy of the renal hilum as easy if only one artery with a late bifurcation (lateral for the IVC on the right side, and more than 2 cm from the aorta on the left side) and one vein were present (Type 1L and 1R). Early bifurcation of the artery (behind IVC on the right side for Type 2R, and less than 2 cm from the aorta on the left side for type 2L) and more than one vein (Type 3R and 3L) or artery (Type 4R and 4L) was defined as a complex anatomy.

Patient factors such as age, BMI, previous renal surgery, inflammation (pyelonephritis or pancreatitis), radiation and immunotherapy were collected from patients’ medical records, as well as vascular anatomy and patterns observed in CT scans. Surgical factors such as intraoperative bleeding during RHC, surgeon, and approach were recorded and analysed using descriptive statistics.

We used SPSS software, including independent samples t-test, one-way ANOVA and Bonferroni multiple comparisons to identify the correlation of these factors and vascular patterns with THC. Additionally, we used stepwise multivariable regression models to assess the predictors, and statistical significance was set at p < 0.05.

## Results

Population and surgical features are summarised in Table 1. The cohort comprised 72% males and had a median age of 67.5 years and an interquartile range (IQR) of 15. Most of the patients did not have previous kidney surgery, inflammation, radiotherapy or immunotherapy. The operations were distributed between three surgeons in a 1:1.8:1.5 manner and only 18.3% of RAPN were performed via a retroperitoneal approach.

**Table 1:** Population demographics and surgical features

Variable	Overall (n = 60)	Variable	Overall (n = 60)
*Age (years)* Median [IQR]	67.5 (15)	*Vascular patterns* Type 1R Type 2R Type 3R Type 4R Type 1L Type 2L Type 3L Type 4 L	16 (26.7%) 6 (10%) 6 (10%) 3 (5%) 18 (30%) 2 (3.3%) 2 (3.3%) 7 (11.7%)
*Gender*			
Male	43 (71.7%)		
Female	17 (28.3%)		
*Previous surgery*			
Yes	4 (6.7%)		
No	56 (93.3%)		
*Previous Inflamation*		*Anatomy*	
Yes	2(3.3%)	Easy	34 (56.7%)
No	58 (96.7%)	Complex	26 (43.3%)
*Previous radiotherapy*		*Operator*	
Yes	1 (1.7%)	1	14 (23.3%)
No	59 (98.3)	2	25 (41.7%)
		3	21 (35%)
*Previous imunotherapy*		*Surgical approach*	
Yes	2 (3.3%)	Transperitoneal	49 (81.7%)
No	58 (96.7%)	Retroperitoneal	11 (18.3%)
BMI Median [IQR]	28.1 (6)	*Time to hilum control* *(minutes)*	
*Side*		Median [IQR]	22.7 (17)
Right	31 (51.7%)		
Left	29 (48.3%)		
*Number of arteries*		*Total operative time*	
1	50 (83.3%)	Median [IQR]	133.6 (40)
2	9 (15%)		
3	1 (1.7%)		
*Number of veins*		*Intraoperative bleeding*	
1	53 (88.3%)	Yes	4 (8.7%)
2	7 (11.7%)	No	56 (91.3%)

More than 80% of the patients had only one artery or one vein and the eight vascular patterns are depicted in Fig. 1. Type 1R and 1L were characterised as easy anatomy and represented more than half of the patients (56.7%).

We observed a median THC of 22.7 min, representing 15.1% of TOT. A significant correlation was found between previous renal surgery and longer THC (p = 0.033), as presented in Table 2.

**Table 2:** Factors correlated with time to hilum control and the percent from total operative time

Variable	N	THC		Percent THC/TOT	
		Mean(minutes) */ Correlation**	*p*	Mean (%)*/ Correlation. **	*p*
Gender*			*0.265*		*0.281*
Male	43	24.9		17.2	
Female	17	21.2		17.7	
*Previous surgery*			***0.033***		***0.010***
Yes	56	23.1		16.7	
No	4	35.6		26.5	
*Previous inflammation*					
Yes	58	23.5	*0.129*	17.1	*0.061*
No	2	36.1		27.1	
*Previous radiation*					
Yes	59	23.9	*0.981*	17.4	*0.588*
No	1	24.2		13.1	
*Previous immunotherapy*					
Yes	58	24.1	*0.621*	17.4	*0.673*
No	2	19.9		15.1	
Side*			*0.231*		*0.672*
Right	31	22.2		16.9	
Left	29	25.7		17.7	
Renal arteries*					
1	50	21.9	***0.007***	16.3	***0.051***
2	9	34.7		22.9	
3	1	25.4		17.8	
Renal veins*			*0.482*		*0.446*
1	53	24.3		17.6	
2	7	21.0		15.3	
Anatomy*					
Easy	34	19.7	***0.001***	14.8	***0.002***
Complex	26	29.4		20.7	
Vascular pattern*					
Type 1R Type 2R Type 3R Type 4R Type 1L Type 2L Type 3L Type 4L	16	16.5	***0.02***	13.1	***0.03***
	6	34.5		25.9	
	6	20.6		15.8	
	3	30.7		22.0	
	18	22.5		16.3	
	2	24.3		18.9	
	2	23.8		12.8	
	7	35.1		22.6	
Operator*			*0.753*		*0.220*
1	14	25.9		19.5	
2	25	23.4		17.9	
3	21	23.1		15.2	
Surgical approach*			*0.387*		*0.062*
Transperitoneal	49	23.3		16.5	
Retroperitoneal	11	26.6		21.1	
Intraoperative bleeding*			***0.05***		*0.242*
Yes	56	23.1		17.2	
No	4	34.6		18.7	
Age**	60	.085	*0.516*	0.016	*0.902*
BMI**	60	.089	*0.499*	0.086	*0.514*

Bold and bold Italic for "*p*" means *p*<0.05

*THC* time to hilum control, *TOT* total operative time, *BMI* body mass index

*ANOVA/t-test

**Pearson

Having a complex vascular anatomy (p = 0.001) with a particular vascular pattern (p = 0.02) and more than one kidney artery (p = 0.007) correlated with longer THC. The Bonferroni multiple comparisons of the vascular patterns demonstrated that type 2R (renal artery bifurcation behind the IVC) and 4L (more than one main artery on the left side) were significantly associated with longer THC compared to the other vascular patterns (Table 3).

**Table 3:** Bonferroni multiple comparisons having time to hilum control as the dependent variable

(I) Vascular pattern	(J) Vascular pattern	Mean difference (I-J)	Std. error	Sig	95% Confidence Interval	
					Lower bound	Upper bound
Type 1R	**Type 2R**	−18.0135	4.7379	**0.011**	−33.616	−2.411
	Type 3R	−4.0608	4.7379	1.000	−19.664	11.542
	Type 4R	−14.1913	6.2268	0.750	−34.697	6.315
	Type 1L	−5.9756	3.4006	1.000	−17.174	5.223
	Type 2L	−7.7469	7.4228	1.000	−32.192	16.698
	Type 3L	−7.3135	7.4228	1.000	−31.758	17.131
	**Type 4L**	−18.5278	4.4850	**0.004**	−33.298	−3.758
Type 2R	Type 1R	18.0135	4.7379	**0.011**	2.411	33.616
	Type 3R	13.9528	5.7141	0.505	−4.865	32.771
	Type 4R	3.8222	6.9983	1.000	−19.225	26.869
	Type 1L	12.0380	4.6655	0.357	−3.327	27.403
	Type 2L	10.2667	8.0809	1.000	−16.346	36.879
	Type 3L	10.7000	8.0809	1.000	−15.912	37.312
	Type 4L	−0.5143	5.5062	1.000	−18.648	17.619
Type 3R	Type 1R	4.0608	4.7379	1.000	−11.542	19.664
	Type 2R	−13.9528	5.7141	0.505	−32.771	4.865
	Type 4R	−10.1306	6.9983	1.000	−33.177	12.916
	Type 1L	−1.9148	4.6655	1.000	−17.279	13.450
	Type 2L	−3.6861	8.0809	1.000	−30.298	22.926
	Type 3L	−3.2528	8.0809	1.000	−29.865	23.360
	Type 4L	−14.4671	5.5062	0.316	−32.600	3.666
Type 4R	Type 1R	14.1913	6.2268	0.750	−6.315	34.697
	Type 2R	−3.8222	6.9983	1.000	−26.869	19.225
	Type 3R	10.1306	6.9983	1.000	−12.916	33.177
	Type 1L	8.2157	6.1719	1.000	−12.110	28.541
	Type 2L	6.4444	9.0348	1.000	−23.309	36.198
	Type 3L	6.8778	9.0348	1.000	−22.876	36.631
	Type 4L	−4.3365	6.8296	1.000	−26.828	18.155
Type 1L	Type 1R	5.9756	3.4006	1.000	−5.223	17.174
	Type 2R	−12.0380	4.6655	0.357	−27.403	3.327
	Type 3R	1.9148	4.6655	1.000	−13.450	17.279
	Type 4R	−8.2157	6.1719	1.000	−28.541	12.110
	Type 2L	−1.7713	7.3768	1.000	−26.065	22.522
	Type 3L	−1.3380	7.3768	1.000	−25.632	22.956
	Type 4L	−12.5522	4.4085	0.176	−27.070	1966
Type 2L	Type 1R	7.7469	7.4228	1.000	−16.698	32.192
	Type 2R	−10.2667	8.0809	1.000	−36.879	16.346
	Type 3R	3.6861	8.0809	1.000	−22.926	30.298
	Type 4R	−6.4444	9.0348	1.000	−36.198	23.309
	Type 1L	1.7713	7.3768	1.000	−22.522	26.065
	Type 3L	0.4333	9.8971	1.000	−32.160	33.027
	Type 4L	−10.7810	7.9353	1.000	−36.914	15.352
Type 3L	Type 1R	7.3135	7.4228	1.000	−17.131	31.758
	Type 2R	−10.7000	8.0809	1.000	−37.312	15.912
	Type 3R	3.2528	8.0809	1.000	−23.360	29.865
	Type 4R	−6.8778	9.0348	1.000	−36.631	22.876
	Type 1L	1.3380	7.3768	1.000	−22.956	25.632
	Type 2L	−0.4333	9.8971	1.000	−33.027	32.160
	Type 4L	−11.2143	7.9353	1.000	−37.347	14.918
Type 4L	Type 1R	18.5278	4.4850	**0.004**	3.758	33.298
	Type 2R	0.5143	5.5062	1.000	−17.619	18.648
	Type 3R	14.4671	5.5062	0.316	−3.666	32.600
	Type 4R	4.3365	6.8296	1.000	−18.155	26.828
	Type 1L	12.5522	4.4085	0.176	−1.966	27.070
	Type 2L	10.7810	7.9353	1.000	−15.352	36.914
	Type 3L	11.2143	7.9353	1.000	−14.918	37.347

Bold and bold Italic for "*p*" means *p*<0.05

Intraoperative bleeding during RHC was low (8.7%) and correlated with longer THC (p = 0.05), though no significant difference was found between surgeons (p = 0.753) or surgical approach (transperitoneal vs. retroperitoneal, p = 0.87).

After stepwise regression, the best model identified to predict THC included anatomical complexity and previous surgery (R-square = 0.281, p = 0.006), as shown in Table 4.

**Table 4:** Stepwise regression analyses for warm ischemia time, excision time, reconstruction time and intermediate time

Model summary									
Model	R	R square	Adjusted R square	Std. error of the estimate	Change statistics				
					R square change	F change	df1	df2	Sig. F Change
1	0.423^a^	0.179	0.165	628.490	0.179	12.673	1	58	< 0.001
2	0.530^b^	0.281	0.256	593.283	0.102	8.088	1	57	0.006

^a^Predictors: (Constant), Anatomy Complexity

^b^Predictors: (Constant), Anatomy Complexity, Previous Surgery

## Discussion

Our present study identified, on CT scan, several patterns according to the disposition of the vascular structures in the renal hilum. The analyses revealed which patterns correlate to a more complex anatomy and longer THC.

To the best of our knowledge, no data are available on intraoperative video documentation review for RHC assessment during RAPN. Previous studies have assessed the importance of SVR. For example, De Backer et al. [5] considered 100 RAPNs and showed that surgical phase duration can be correlated with certain clinical outcomes. Kim et al. [6] tested SVR to assess the Mayo adhesive probability score and perirenal fat dissection time. Additionally, our group used SVR to evaluate WIT during RAPN and its impact on surgical margins and complication rate, in a previous study [7].

We found THC to be a relatively short portion (15%) of the TOT. For the present study, TOT depicts the total console surgery time as the time used to achieve pneumoperitoneum or docking the robot were not taken into account. De Backer et al. [5] also found a short time to hilum control with a median of 16 (±11.5) minutes, as compared to 22 min in our study. This could be due to including patients with redo RAPN and complex vascular anatomy in our cohort. Of the 100 patients in the cohort of De Backer et al., 77 procedures were performed offclamp that requires less dissection of all the hilar structures.

Previous renal surgery was registered in four of the patients who underwent a redo RAPN for tumour resection elsewhere in the kidney than previous resection bed. It correlated to longer THC as more fibrosis around the vascular structures in the hilum needed to be dissected in order to achieve RHC. No statistical correlation was found between longer THC and previous inflammation like pyelonephritis or pancreatitis, previous radiotherapy, or previous immunotherapy. This finding could be biased by the low number of patients (only one or two) who did not have such previous conditions. Though previous abdominal surgery poses surgical challenges for PN [8], several studies identified no impact on the outcomes of RAPN [9, 10]. Redo RAPN is considered an effective approach [11], and evidence indicates good feasibility and safety, though it is a challenging procedure [12]. One challenge is RHC and it makes sense that more time is used to dissect the vascular structures, as more fibrosis around the hilar structures is present from previous RHC. Though RAPN remains the preferred option for treating small renal masses, percutaneous cryoablation is a valid alternative, particularly for challenging patients, as shown by Iossa et al. [13]. Two out of four patients who underwent redo RAPN were performed retroperitoneally. This could have biased our results together with a disproportioned percent of only 18% of the patients performed a retroperitoneally approach. This could also explain why we registered longer THC in the retroperitoneal group.

The anatomy of the renal vascular system and its application to PN was initially presented by Graves in 1954 with a special focus on segmental branching of the renal artery and its role in achieving selective ischemia [14]. Later, Trivedi et al. [15] identified six vascular branching patterns of segmental renal artery in an anatomical study and stated that knowledge of these patterns is essential to effective surgical planning in cases of PN and preventing complications. Clamping the segmental renal artery instead of the main renal artery during PN is a promising technique to decrease WIT, and understanding vascular branching patterns is essential for an appropriate hilar approach. However, selective or superselective clamping do not provide better renal function preservation than renal artery conventional clamping, as shown in systematic reviews [16] and randomized controlled trials [17]. This places doubt on the benefit of these techniques which additionally has higher risk of bleeding. To the best of our knowledge, no data are available on renal vascular anatomy with focus on conventional clamping, and our study identified vascular patterns that help for a more detailed understanding of renal vascular anatomy including cases of renal hilar masses, where RAPN is a promising technique [18].

Multiple authors have described the arrangement of renal hilar structures, through anatomical studies, identifying several patterns [4, 19] which included the renal pelvis into their classifications. We used vascular structures in our classification as only the vein and artery are dissected during the RHC for PN. We identified eight patterns, four for each side, including both the normal structure and variations. A complete understanding of underlying normal and aberrant renal anatomy, coupled with patient- and tumour-specific anatomical characteristics, represents the foundation for proper preoperative surgical planning for PN [20]. We determined that having more than one renal artery requires longer THC as more time is expected to be used to dissect all the arteries to be clamped. In our classification, we did not include small accessory arteries that did not need to be clamped during WIT. An early bifurcation of the renal artery on both sides but especially on the right side, as the artery passes behind the IVC made the dissection of the main artery more difficult, and longer THC was observed.

We did not register a statistical significance between the three surgeons. Larcher et al. [21] indicated that RAPN outcomes might be affected by surgeon experience by shortening WIT and lowering the complication rate. The inhomogeneous surgical experience among the surgical team members and the small sample size caused us to evaluate the surgeons as an independent variable, instead of surgeon’s expertise, and that may have biased our results. Intraoperative bleeding rate was low during RHC and correlated with longer THC as more time was needed to control the bleeding before controlling the renal hilum.

Some patient characteristics and comorbidities were not recorded due to the retrospective design of this study. The study was not blinded because SVR requires a trained kidney surgeon to identify the specific steps of the procedure and no interobserver validation of pattern assignment was attempted. The vascular patterns identified in our study are valuable for cases when clamping of the main artery is chosen; for selective clamping, different intrarenal anatomical patterns should be considered. Despite these limitations, our results can be used to optimise preoperative surgical planning for RAPN and provide a basis for developing a hilum complexity score in the future.

## Conclusion

THC represents a relatively short part of total console time during RAPN. Previous renal surgery and a complex vascular pattern with artery bifurcation behind IVC on the right side and more than one main renal artery on the left side can lead to longer THC. A detailed understanding of renal vascular patterns can provide patient-specific surgical planning and optimise surgical strategies for RAPN.

## Data Availability

No datasets were generated or analysed during the current study.
